# rSEM: System-Entropy-Measure-Guided Routing Algorithm for Industrial Wireless Sensor Networks

**DOI:** 10.3390/s22218291

**Published:** 2022-10-29

**Authors:** Xiaoxiong Xiong, Chao Dong, Kai Niu

**Affiliations:** Key Laboratory of Universal Wireless Communications, Beijing University of Posts and Telecommunications, Beijing 100876, China

**Keywords:** system entropy, routing, energy saving, reducing the delay

## Abstract

In this paper, a new system entropy measure is used to optimize the routing algorithm in power consumption. We introduce the system entropy measure into the problem of industrial wireless sensor networks (iWSNs) routing and propose a high-performance routing algorithm guided by the system entropy measure (rSEM). Based on the cluster iWSNs architecture, the rSEM selects the cluster heads and cluster member nodes successively, according to the system entropy measure, and constructs the iWSNs with the minimum system entropy. The method of the cluster head selection is traversal, while the method of the cluster member selection is a greedy algorithm to reduce the complexity. The experiments show that the power consumption of the iWSNs generated by the rSEM is in the same order of magnitude as that of Dijkstra in both 2D and 3D scenarios. In addition, the delay of the rSEM is slightly higher than that of LEACH. Therefore, the rSEM is suitable for networks that are sensitive to both the delay and power consumption. The rSEM puts forward a new idea for the design of routing for the next-generation iWSNs, which improves the overall network performance according to the network topology, instead of relying on the power consumption or delay performance only.

## 1. Introduction

With the advent of the 5G technology era [1], the industrial Internet of Things system will undergo profound changes, among which the most prominent feature is the more compact layout and larger number of sensors [2]. How to manage such large iWSNs has always been a hot topic in the industrial network academia, and routing is the key technology. This technology comprehensively considers the node location and geographical topology to develop the most suitable link between the nodes in the current network. Obviously, most routing algorithms are self-adaptive [3] in order to adapt to different scenarios. However, completing the self-adaptation will inevitably bring some problems. Optimizing a network by a single measure tends to lead to two extremes: extravagant delay or energy consumption.

Among the algorithms that optimize the network from the perspective of power minimization or total path minimization, the Dijkstra algorithm [4,5] is the most representative one to apply to power-sensitive iWSNs. The shortest path from the single-source algorithm Dijkstra can search the shortest path from point to point through the traversal. However, the delay disadvantage of this algorithm will be amplified in some special scenarios, such as the 5G high-frequency channel simulated in the experiment in this paper. This is because the high-frequency attenuation is serious; the Dijkstra algorithm may carry out the signal relay frequently in order to minimize the power consumption, which leads to more hops and an excessive delay.

In order to balance the relationship between the power consumption and delay, LEACH [6,7] was proposed as a two-hop hierarchical cluster routing algorithm, which effectively compressed the average number of hops lower than two to achieve a trade-off between the energy consumption and the delay. This two-hop restriction is very suitable for delay-sensitive iWSNs. In addition, in order to balance the load, the round-robin mechanism of the cluster heads is introduced to ensure the load balance of each node. However, in the process of rotating cluster heads, some isolated or severely off-center nodes [8,9,10], in the sense of the geographical location, also have the chance to become cluster heads, which is undesirable in a practical application.

Obviously, the performance of the network is affected by the topology. The reason why it will go to the two extremes is the lack of a systematic network measure. The concept of system entropy is proposed in [11], which measures the comprehensive performance of a network by its topology. There is only one variable, i.e., the Laplace matrix, in the expression of the system entropy measure, and the network topology matrix is used to evaluate the network performance in a more abstract way. The system entropy measure points out a new way for a future network.

This paper introduces the system entropy measure into the problem of routing and proposes a high-performance routing algorithm based on the system entropy measure (rSEM). The algorithm is also used for routing in a two-hop cluster network. One of the differences between LEACH and the rSEM is that once the cluster head is selected, it does not change, and so is the cluster member (the round-robin mechanism of cluster heads is abandoned). Moreover, the selection of cluster heads by the rSEM is not random. Firstly, a group of nodes suitable for a cluster head is selected by minimizing the system entropy measure. So, the high-layer network composed of cluster head nodes and a sinknode has the minimum system entropy. Then, according to the system entropy measure, the cluster head suitable for the remaining cluster member nodes (in the basic layer) is selected, and the final result also meets the effect of the minimum system entropy measure. The experiments show that the power consumption of an iWSN generated by the rSEM is moderately higher but in the same order of magnitude as that of the Dijkstra algorithm in both 2D and 3D scenarios. In addition, the experiments also show that the hops the network generated by the rSEM are slightly higher than the LEACH algorithm but strictly lower than two hops. Therefore, the rSEM is suitable for networks that are sensitive to both a delay and power consumption. The rSEM algorithm is a reference for the routing algorithm design of the next-generation iWSNs.

The notations used in this paper is as follows: capital bold letters denote matrices, and capital italics generally denote scalars or sets. The right superscript of a variable or matrix represents a description of the properties, and the right superscript of $R^{m\times n}$ represents the set R of dimension m×n. $L_{\left\{i,j\right\}}$ represents the element at the *i*-th row and the *j*-th column of L. The matrix $L_{s}$ or $L_{s,i}$ with the right subscript represents the L matrix of some round in the multi-cycle operation.

The organization of this paper is as follows: Section 2 introduces the simulation scenario and previous related work; Section 3 introduces the rSEM algorithm in detail. Section 4 introduces the experimental configuration and experimental results. Section 5 brings the conclusion.

## 2. System Model and Previous Work

The industrial scene and the layout of nodes can be abstracted as the process of scattering points on the plane (or in the cube). When the geographical location of nodes is fixed, the next step should be to reasonably plan the connection relationship between the nodes to ensure the efficient operation of the network. When the total energy loss becomes the focus, the Dijkstra algorithm is often used to complete the optimal organization of the network.

**Figure 1:**
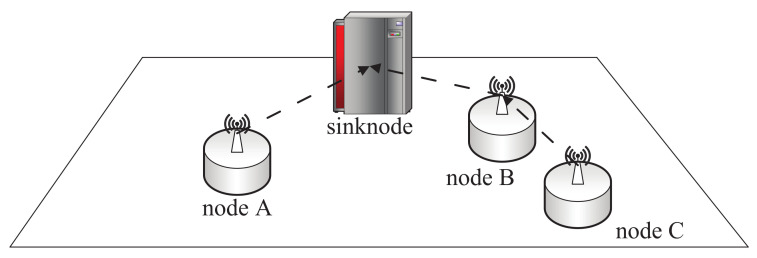
The simulation scenario discussed in this paper.

Figure 1 shows a schematic of a two-dimensional factory in the form of a single-cell scenario used in communication. The only sinknode performs the data aggregation. All the nodes in the factory need to report their data to the sinknode. There are two ways for a sensor to report: The first is to report directly to the sinknode, as node A. The second is node C in the figure, where data are transferred to the sinknode via node B (sometimes more than two hops). The task of the routing algorithm is to search a path to the sinknode for each sensor. Specifically, the routing algorithm needs to let the data of the sensor reach the sinknode directly (like node A), or reach the sinknode through the appropriate relay (like node C). In this paper, we define a successful communication need as an SNR as follows:   

(1) $$SNR=20\log_{10}\left(\frac{E\cdot h\cdot Pathloss}{N_{0}}\right)>sensitivity\left[dB\right],$$

where *E*, *h*, Pathloss, and $N_{0}$ represent the transmit power of the sensor, the channel small-scale fading, the channel path loss, and the additive white Gaussian noise power of the channel, respectively. Expression (1) shows that the premise of successful communication is that the received SNR is higher than the received sensitivity. The channel small-scale fading *h* here is Rayleigh fast fading, i.e., h∼N(0,1).

The Dijkstra algorithm can realize the point-to-point routing of the whole network according to the minimum cost of a parameter. Therefore, Dijkstra is a routing algorithm suitable for power-sensitive networks and provides the optimal low-power routing topology of the whole network. However, there is always a log conversion relationship between the distance and the path loss, which makes the relationship between the distance and the path loss not linear. As a direct result of this phenomenon, the point-to-point path with the minimum power consumption in the network is with relay, not directly. Once the relay node has a large degree, the information processing at the hub with a high degree will be delayed, which will cause the network congestion.

The LEACH algorithm comprehensively considers the above problem and can compress the hops of the network to less than 2 [6,7]. Therefore, LEACH is a routing algorithm suitable for delay-sensitive networks. In order to balance the forwarding efficiency of the network and the power consumption of nodes, the LEACH algorithm adopts a hierarchical structure (cluster structure), that is, there are cluster heads and cluster member nodes in the network. Nodes can report to the sinknode through the cluster head or directly. The selection of the two cases is based on the shortest path or the minimum power consumption. In addition, in order to prolong the life of the network, the cluster head nodes in the network are round-robin periodically, which may lead to an increase in the network power consumption when an isolated node [8,9] becomes a cluster head. Each node *i* in the network generates a random number between 0 and 1. If the number of a node is less than the threshold T(i), the node becomes a cluster head for the current slot. T(i) can be computed as shown in the following:

(2) $$T\left(i\right)=\left\{\begin{array}{cccc} \; & \frac{Per}{1-Per\times \left(r\times \;\mathrm{mod}\;\left(\frac{1}{Per}\right)\right)} & , & i\in \zeta \\ \; & 0 & , & i\notin \zeta \end{array}\right.,$$

where *r* is the index of current slot, Per is the desired percentage of cluster head in the slot, and *n* is the number of total nodes in the network. The set ζ is the set that includes nodes that have not become cluster head in the final 1/Per slots.

In order to avoid such isolated nodes becoming cluster heads, Ref. [10] proposed the RED\_LEACH algorithm to improve LEACH. The specific improvement is that the distance from the cluster head to the sinknode is lower than the average distance from all the sensors to the sinknode.

The location of the cluster heads is closer to the sinknode, which can obviously improve the power performance of LEACH. However, the nature of cluster head elections is still randomization. In order to further improve the power performance of the LEACH algorithm, some optimization methods are needed to replace the LEACH random cluster head election.

The advantages and disadvantages of the above two algorithms are self-evident. The Dijkstra algorithm focuses on energy consumption, while LEACH focuses on load balancing and the network delay. This paper focuses on the problem of how to reduce the power consumption as much as possible on the premise of ensuring a low delay. Therefore, the rSEM algorithm proposed in this paper is suitable for networks sensitive to both the delay and power consumption, which also accords with the future industrial network development trend [1]. In the next section, the details of the rSEM algorithm are presented.

## 3. rSEM Algorithm

Considering the needs of the efficient transfer of data and energy saving, this section will explore the routing based on system entropy suitable for future industrial networks. The system entropy measure is a network performance measure based on topology. In addition, the topology structure of the network inherently contains various advantages or disadvantages in power consumption and delay performance. Therefore, the optimization of the network based on the system entropy measure is expected to reduce the network power consumption from the perspective of topology. At the same time, from the experimental results, the rSEM algorithm also outperforms the Dijkstra algorithm in delay.

Firstly, the rSEM algorithm uses Laplace matrix L to describe the network topology. The elements $L_{\left\{i,i\right\}}$ at the diagonal of the Laplace matrix represent degrees, and the elements outside the diagonal $L_{\left\{i,j\right\}}$s are represented by negative capacity values as

(3) $$L_{\left\{i,j\right\}}=-\log_{2}\left(\frac{Pathloss\left(i,j\right)}{N_{0}}+1\right)$$

where $Pathloss\left(i,j\right)$ represents the path loss between node *i* and node *j*, $N_{0}$ is the noise power. If there is no connection between two points in the routing, $L_{\left\{i,j\right\}}=0$.

The expression of system entropy is as follows [12,13]:

(4) $$H^{sys}\left(L\right)=\frac{1}{1-\alpha }\log_{2}\left[tr\left({\left(L\right)}^{\alpha }\right)\right]$$

where α represents the parameter of system entropy and its value is 2, which is different from the description in the literature [11]. When using the capacity as a path metric, an α of less than one leads to the system entropy in complex form.

The system entropy-based routing uses the hierarchical routing architecture within 2 hops. Sinknode selects cluster heads, and cluster member nodes select cluster head nodes connecting to themselves to achieve network optimization.

Next, the specific network optimization steps based on system entropy theory are introduced. Assuming that *N* nodes and 1 sinknode are at known positions. Firstly, $N^{ch}$ (preset) cluster heads needs to be selected from *N*. Once the number of cluster heads is determined, $N^{ch}$ nodes that are most suitable to be cluster heads can be determined through traversal, and the selection method is as follows [11]:

(5) $$\underset{L_{i}^{ch}\in \Omega }{L^{ch}=\arg\min}\;\left[H^{sys}\left(L_{i}^{ch}\right)\right]$$

where Ω represents all the cases that need to be considered in the problem, obviously, $\left|\Omega \right|=\left(\begin{array}{c} N\\ N^{ch} \end{array}\right)$, where $\left(\begin{array}{c} n\\ m \end{array}\right)$ is the combinatorial function. $L_{i}^{ch}\in R^{\left(N^{ch}+1\right)\times \left(N^{ch}+1\right)}$ represents the topology of the network composed of some $N^{ch}$ nodes and the sinknode. If the cluster head only needs to report to the sinknode, all the elements on the diagonal are 1 except that of the sinknode.

The cluster head selection method is similar to the maximum likelihood traversal method, and all cases are exhaustively compared to find $N^{ch}$ cluster heads corresponding to the case with the minimum system entropy. After the cluster heads selection, the cluster member nodes will choose cluster heads, or connect directly to the sinknode. Obviously, if all the cases were tried, the algorithm has to traverse ${\left(N-N^{ch}\right)}^{N^{ch}+1}$ cases in total. In order to simplify the algorithm better, the parameter *v* can be set to describe the number of potential cluster heads considering by each member node. The definition of potential cluster heads is by minimum distance: member nodes consider only the nearest *v* cluster heads (or v−1 cluster heads and the sinknode). Member nodes in this step are added one by one. For *s*-th ($s=1,\ldots ,N-N^{ch}$) selection, only $\left(N-N^{ch}-\left(s-1\right)\right)\times v$ cases need to be considered, where s−1 is the number of cluster member nodes that have been connected to the network. The *s*-th selection is

(6) $$L_{s}=\underset{L_{s,i}\in \Psi }{\arg\min}\;\left[H^{sys}\left(L_{s,i}\right)\right],$$

where Ψ represents the set formed by the above $\left(N-N^{ch}-\left(s-1\right)\right)\times v$ cases where each unconnected member node has *v* cases. $L_{s,i}\in R^{\left(N^{ch}+s+1\right)\times \left(N^{ch}+s+1\right)}$ is obtained from $L_{s-1}$ by extending it by one dimension and adding the corresponding degrees and connections. When s=1, the algorithm operates the extending and adding on the matrix $L^{ch}$. The specific algorithm steps are in Algorithm 1.
**Algorithm 1:** Pseudocode of the rSEM Algorithm**Input:** *N*, $N^{ch}$, *v*, location of the nodes    **Output: **L      1: According to $\left(\begin{array}{c} N\\ N^{ch} \end{array}\right)$ cases, the set Ω is obtained;      2: Selecting the $N^{ch}$ cluster heads from the set Ω as (5) to get $L^{ch}$;      3: Let $L_{0}$ = $L^{ch}$;      4: For every remaining $N-N^{ch}$ member node, *v* candidate cluster head nodes are selected based on the minimum distance principle for it;      5: According to the above $(N-N^{ch})\times v$ cases, the initial set Ψ is obtained;      6: **for**s=1 to $N-N^{ch}$
**do**      7:    Selecting a member node from current Ψ as (6);      8:    Adding this member node to $L_{s-1}$ to get $L_{s}$;      9:    Deleting the *v* cases about this member node to get an updated Ψ;    10: **end for**    11: Let L = $L_{N-N^{ch}}$;    12: **return **
L


Obviously, the usage of the parameter *v* and the greedy strategy will lead to a reduction in the discussed cases, and the case with the minimum system entropy may not be included. Therefore, if we want to obtain the minimum entropy of the network system, we need to traverse more cases, that is, a larger *v*. In the experiment of this paper, v=2 is used, which is a trade-off between complexity and performance. The trade-off not only ensures enough traversal cases but also ensures the complexity of the algorithm within an acceptable range.

## 4. Results

This section will introduce the experimental results about the system entropy routing method and mainly focuses on the power consumption. All the simulations in this paper are carried out in MATLAB 2020, and the random functions of the software are used to realize the scatter process described, the generation of white noise, and the generation of small-scale fading. The measurement of the network power consumption is described as follows: After the routing algorithm searches the path of the network, each sensor reports its data to the sinknode. Through a large number of simulations for the same routing scheme and in the same topology, the transmitting power is obtained under the premise of satisfying a certain successful connection rate. Obviously, the smaller the power is, the more reasonable the routing is and the lower the power consumption is. The required successful connection rate in this experiment is 0.9 (like the targeted outage probability in [14]). Once the routing algorithm has determined the network connection, all that remains is to generate a large number of small-scale random fading numbers to test the successful connection rate of the current network. By appropriately increasing or decreasing the transmitting power of the sensor, the average connectivity probability of the network converges to the required successful connection rate 0.9. The final successful connection rate $P_{f}$ error tolerated by the experiment is 0.0001, i.e., the final successful connection rate satisfies $\left|P_{f}-0.9\right|\leq 0.0001$. In addition, the connection rate for each transmitting power comes from the average result of 10,000 independent Rayleigh fast fading *h*. The receiving sensitivity of a sensor is 2 dB. The noise power here is −104 dBm (thermal noise within 1 MHz bandwidth). In the experiment, the number of cluster heads is assumed to account for 50%. The network consists of 16 nodes and 1 sinknode. In addition, four slots are preset for LEACH and RED\_LEACH, which ensures the fairness.

The millimeter-wave frequency band in the 5G communication scenario is discussed, and the carrier frequency is $f_{c}=30$ GHz. Therefore, the following path loss model is used [15]:

(7) $$PL=\left\{\begin{array}{ll} \;32.4+\;21\log_{10}(\mathrm{distence}\;\;\;\left[\;\;\;m\;\;\;\right]\;\;\;)\;+\;20\log_{10}(f_{c}\;\;\;\left[\;\;\;\mathrm{GHz}\;\;\;\right]\;\;\;)\;, & \mathrm{LOS}\\ \;22.4+35.3\log_{10}(\mathrm{distence}\;\;\;\left[\;\;\;m\;\;\;\right]\;\;\;)\;+\;21.3\log_{10}(f_{c}\;\;\;\left[\;\;\;\mathrm{GHz}\;\;\;\right]\;\;\;), & \mathrm{NLOS} \end{array}\right.\;\;,\;\;\mathrm{distence}>10\;m$$

Among them, the LOS and NLOS are included. Considering that the rSEM algorithm is designed to adapt to the future smart factory, the production line with a complex structure and composition will inevitably block electromagnetic waves. In this case, the LOS and NLOS must coexist. In this experiment, the LOS and NLOS characteristics of each wireless channel are established when the topology is formed, and the ratio of the LOS to the NLOS is preset to be 9:1 under the feature of a relatively close distance. The LOS/NLOS characteristic of each connection is generated by the Bernoulli distribution of a 0.9 success probability.

**Figure 2:**
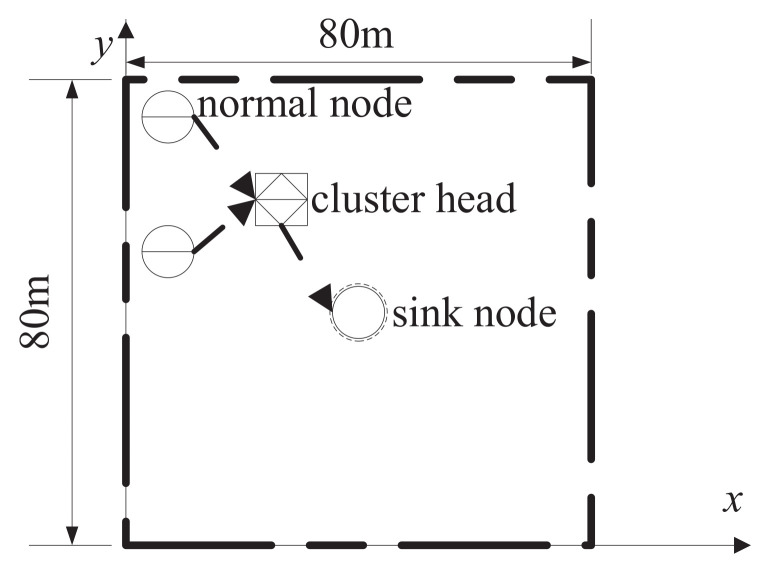
Schematic diagram of cluster structure iWSNs in 2D factory.

In the first experiment, the scatter method of the sensor nodes is to randomly scatter 16 nodes in the 2D range of 80m×80m, and the sinknode is located in the position of (40m,40m), as shown in Figure 2. Considering the use of the large-scale transmission model of the 3GPP above, the pairwise distance of the 16 sensor nodes should be maintained at more than 10 m. If the single scatter point fails to meet this condition, the scatter point will be re-scattered until it is satisfied.

**Figure 3:**
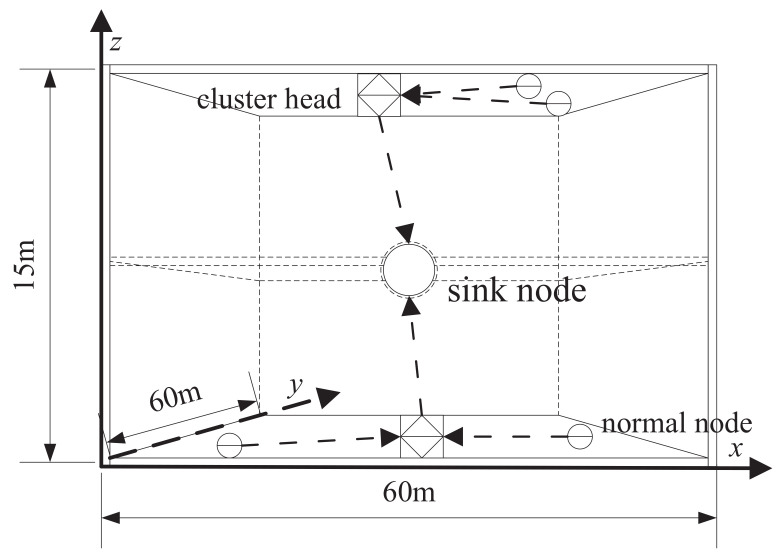
Schematic diagram of cluster structure iWSNs in 3D factory.

The second experiment will simulate a scenario of a two-floor factory building, aiming to simulate a scenario with more dense nodes. The 10m interval between nodes is also maintained from the perspective of 3D. The height of the two-story factory is 15m, and the size of each floor is 60m×60m. The scatter method of the sensor nodes is as follows: Firstly, sprinkle eight nodes randomly in the 60m×60m first floor of the factory, and then sprinkle eight nodes randomly in the 60m×60m second floor of the factory. The sinknode is located at (30m,30m,7.5m), as shown in Figure 3.

**Figure 4:**
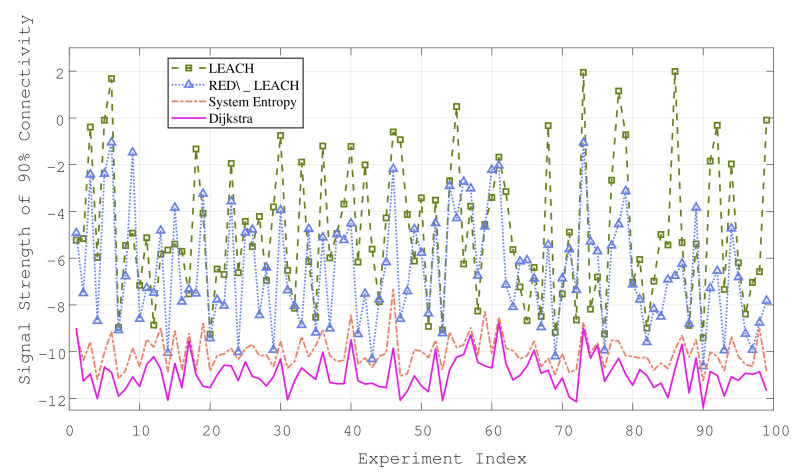
Simulation results in 2D factory scenario.

**Figure 5:**
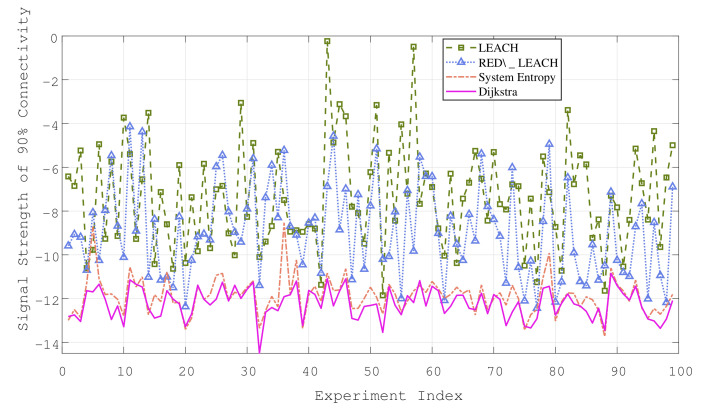
Simulation results in 3D factory scenario.

Figure 4 and Figure 5 show the average power consumption statistics of the 2D and 3D scenarios under 100 groups of different topologies, assuming that there is no signaling conflict between the sensors during the experiment, that is, ALOHA. The ordinate represents the sensor transmit power required under the network connectivity rate of 0.9. As can be seen from the two figures, the oscillation trend of the three curves is consistent. In other words, the topology determines the power consumption level to some extent. For example, there are “isolated nodes” in the sense of distance, which exist in isolation from most node groups. The participation of isolated nodes will inevitably lead to a higher power consumption.

Table 1 shows the statistical results of the power consumption and delay in the 2D and 3D simulation scenarios. Obviously, the 3D denser industrial scene requires a lower average power consumption and fewer hops for the sensor to reach the sinknode. The following conclusions can be drawn from the experimental results: The power consumption of LEACH or RED\_LEACH cannot be in the same order of magnitude as the other two algorithms, and the rSEM outperforms RED\_LEACH in both power consumption and delay. After the statistics of the experimental results, the power consumption of the rSEM algorithm is in the same order of magnitude as that of Dijkstra; this indicates that the system entropy measure can optimize the selection of the cluster heads and cluster members by evaluating the topology. The system entropy measure ensures that the power consumption performance of the rSEM can approximate the optimal routing algorithm under the limit of two hops. Meanwhile, compared with the Dijkstra algorithm in the 2D and 3D scenarios, the delay of the rSEM is reduced by 29.8% and 18.4%, respectively. In addition, the rSEM only needs 5% more delay than LEACH to achieve the above performance. Therefore, the rSEM is the most suitable routing algorithm for the time-delay and power consumption dual-sensitive networks among the four methods simulated in this paper.

The results of the power consumption and delay show that the rSEM algorithm with the network topology as a variable can comprehensively optimize the network performance, reduce the power consumption, and improve the network efficiency. From the experimental results, the rSEM routing algorithm is more suitable for the future “power and delay sensitive networks”.

## 5. Conclusions

This paper introduces the system entropy measure into the problem of routing and proposes a high-power performance rSEM algorithm. In a two-hop cluster network architecture, the cluster head selection and cluster member selection are carried out based on the principle of the minimum system entropy measure. The experiments show that the power consumption of the network generated by the rSEM can reach the same magnitude as Dijkstra’s algorithm. Moreover, the delay performance of the rSEM algorithm is very close to that of the LEACH algorithm. The rSEM algorithm provides an optimization idea for the routing design of the next-generation industrial network from the perspective of the network topology and improves the comprehensive performance of the network. Therefore, the rSEM provides a solution for 5G industrial networks with “time-delay and power consumption dual sensitivity”.

## Figures and Tables

**Table 1:** Comparison of power consumption and delay performance among rSEM, LEACH, and Dijkstra.

	2D		3D	
	Power (dBm)	Delay (hops)	Power (dBm)	Delay (hops)
Dijkstra	−10.9498	2.1332	−12.2281	1.8245
rSEM	−10.0275	1.4968	−11.8600	1.4886
RED\_LEACH	−6.5284	1.5878	−8.9027	1.5278
LEACH	−4.9473	1.4362	−7.3625	1.4141

## Data Availability

Not applicable.

## Abbreviations

The following abbreviations are used in this manuscript:

iWSNs	industrial wireless sensor networks
LEACH	low-energy adaptive clustering hierarchy
5G	fifth generation
SNR	signal-to-noise ratio
LOS	line of sight
NLOS	non-line of sight

## References

[1] Shafi M., Molisch A.F., Smith P.J., Haustein T., Zhu P., De Silva P., Tufvesson F., Benjebbour A., Wunder G. (2017). 5G: A tutorial overview of standards trials challenges deployment and practice. IEEE J. Ser. Areas Commun..

[2] Akpakwu G.A., Silva B.J., Hancke G.P., Abu-Mahfouz A.M. (2018). A Survey on 5G Networks for the Internet of Things: Communication Technologies and Challenges. IEEE Access.

[3] Glorioso R.M., Grueneich G.R., McElroy D. Adaptive routing in a large communications network. Proceedings of the 1970 IEEE Symposium on Adaptive Processes (9th) Decision and Control.

[4] Dijkstra E. (1959). A note on two problems in connexion with graphs. Numer. Math..

[5] Alpert C.J., Hu T.C., Huang J.H., Kahng A.B. A direct combination of the Prim and Dijkstra constructions for improved performance-driven global routing. Proceedings of the 1993 IEEE International Symposium on Circuits and Systems.

[6] Singh S.K., Singh M.P., Singh D.K. (2010). A Survey of EnergyEfficient Hierarchical Cluster-based Routing in Wireless Sensor Networks. Int. J. Adv. Netw. Appl. (IJANA).

[7] Wu X., Wang S. Performance Comparison of LEACH and LEACH-C Protocols by NS2. Proceedings of the 2010 Ninth International Symposium on Distributed Computing and Applications to Business, Engineering and Science.

[8] Zhang F., Yin Z., Gu A., Li Y., Liu H. Research on Simulation of Cluster Routing Protocol for Industrial Wireless Sensor Networks. Proceedings of the 2018 IEEE 4th International Conference on Computer and Communications (ICCC).

[9] Wen T., Hu Y., Zhang B., Long Z. Research on LEACH Routing Algorithm Based on Fuzzy Logic. Proceedings of the 2020 IEEE 4th Conference on Energy Internet and Energy System Integration (EI2).

[10] Chit T.A., Zar K.T. Lifetime Improvement of Wireless Sensor Network using Residual Energy and Distance Parameters on LEACH Protocol. Proceedings of the 2018 18th International Symposium on Communications and Information Technologies (ISCIT).

[11] Dong C., Xiong X., Xue Q., Zhang Z., Niu K., Zhang P. (2022). A Survey on the Network Models applied in the Industrial Network Optimization. arXiv.

[12] Cover T.M., Thomas J.A. (1991). Elements of Information Theory.

[13] Yu S., Wickstrøm K., Jenssen R., Príncipe J.C. (2021). Understanding Convolutional Neural Networks With Information Theory: An Initial Exploration. IEEE Trans. Neural Netw. Learn. Syst..

[14] Huang C., Zhang R., Cui S. (2014). Optimal Power Allocation for Wireless Sensor Networks with Outage Constraint. IEEE Wirel. Commun. Lett..

[15] (2019). (V16.0.0): Study on Channel Model for Frequencies from 0.5 to 100 GHz.

